# Deep learning based banana plant detection and counting using high-resolution red-green-blue (RGB) images collected from unmanned aerial vehicle (UAV)

**DOI:** 10.1371/journal.pone.0223906

**Published:** 2019-10-17

**Authors:** Bipul Neupane, Teerayut Horanont, Nguyen Duy Hung

**Affiliations:** School of Information, Computer and Communication Technology, Sirindhorn International Institute of Technology, Pathum Thani, Thailand; University of Maryland at College Park, UNITED STATES

## Abstract

The production of banana—one of the highly consumed fruits—is highly affected due to loss of certain number of banana plants in an early phase of vegetation. This affects the ability of farmers to forecast and estimate the production of banana. In this paper, we propose a deep learning (DL) based method to precisely detect and count banana plants on a farm exclusive of other plants, using high resolution RGB aerial images collected from Unmanned Aerial Vehicle (UAV). An attempt to detect the plants on the normal RGB images resulted less than 78.8% recall for our sample images of a commercial banana farm in Thailand. To improve this result, we use three image processing methods—Linear Contrast Stretch, Synthetic Color Transform and Triangular Greenness Index—to enhance the vegetative properties of orthomosaic, generating multiple variants of orthomosaic. Then we separately train a parameter-optimized Convolutional Neural Network (CNN) on manually interpreted banana plant samples seen on each image variants, to produce multiple results of detection on our region of interest. 96.4%, 85.1% and 75.8% of plants were correctly detected on three of our dataset collected from multiple altitude of 40, 50 and 60 meters, of same farm. Further discussion on results obtained from combination of multiple altitude variants are also discussed later in the research, in an attempt to find better altitude combination for data collection from UAV for the detection of banana plants. The results showed that merging the detection results of 40 and 50 meter dataset could detect the plants missed by each other, increasing recall upto 99%.

## Introduction

### Significance of counting banana plants

One of the daily consumed fruits—Banana (genus *Musa*)—has a long history of cultivation and impacts local and global trade. Regardless, this industry have been threatened several times by pests, and viruses like *Banana Bunchy Top Virus* (BBTV) [1]. Diseases like Yellow Sigatoka, Leaf speckle and Cordana, affects the growth of banana plant leaves, and are more active in the hottest and wettest climate, during which the plant tend to grow the most [2]. Thailand being located in a tropical area, is prone to such diseases in banana plants. Despite of following all standards of planting, banana farms in Thailand still face a problem of losing plants within first few months of cultivation. To keep track of productivity, counting the number of plants needs to be automated, rather than tedious manual approach. The objective of this research is focused to automate the count of banana plants during early stage of growth, in which they are prone to lose maturity.

Our strategic approach to obtain precise automation in counting banana plants is to first collect red-green-blue (RGB) aerial images of the affected banana farm, using UAV. These images are then processed to obtain an orthomosaic that provides complete picture of the farm. The idea thereafter is to use deep learning (DL) architecture to learn the shape of banana plants on small tiles of orthomosaic. As the RGB images alone cannot provide high level of accuracy in detection of banana plants, we combine detection results obtained from application of DL on multiple image processing methods, based on their vegetative properties. We also use the same approach on orthomosaic obtained from multiple flight altitudes, to discuss the performance of our strategy on different flight heights and find out better altitude for collecting data. Therefore, our research contribution will be to evaluate the detection performance of each image variant produced from multiple image processing methods, performance of overall method on dataset produced from multiple altitude, and their combinations.

An upper hand of our approach is the use of normal RGB camera, rather than some sophisticated multi-spectral sensors mounted in UAV, which belittles the expense of collecting aerial images. Also compared to satellite photogrammetry, UAV provides cheaper but high resolution large scale images, which directly affects performance of DL. Further, exploitation of vegetative properties of RGB images, will increase the detection performance of DL for banana plants. This could make visible band spectrum comparable to near-infrared (NIR), which is generally used to understand the vegetative indices on images. Another noteworthiness of this work is the combination of capabilities of multiple detection results on multiple image variants, also based on altitude. Furthermore, the automation in counting banana plants, which is the main objective of our work, allows to estimate loss, re-calculate production, and to set a contingency plan for re-plantation in affected areas.

### Related works

Precision agriculture have been substantially blessed by remote sensing applications in last three decades [3, 4]. However, most of the studies are focused on crop monitoring. Much efforts have been made to calculate parameters like vegetation indices, crop height, crop yield, leaf area index, surface soil properties, ground biomass, water stress, canopy height models, and more [5]. The count of any individual crops does not yet make sense to research world, unless they are not large enough and well-spaced during plantation. A banana plant is a tree-like herbaceous plant [6]; tree-like because of its size being the largest of its species, and almost as big as a tree. Banana plants need about 1.5 meters of spacing between each of them [2] during plantation. Being almost big as a tree, the count of vegetative banana plants can be more related to the research area based on forest inventory management, rather than limiting to precision agriculture.

Remote Sensing technologies like satellite and airborne sensors developed rapidly since 1950s [7]. Visual interpretation of aerial images replaced field measurement for forest management since early 1960s [8]. In 1980s, algorithms for digital imagery based automation in tree detection and delineation were demonstrated by [9], where the author used local maxima of brightness in smoothed aerial images to locate the centre of a tree crown. Algorithms like valley-following and rule-based algorithm [10], multiple scale analysis [11] and model-based template matching techniques [12] were used in the 90’s to detect coniferous tree crowns. Much effort have been made to delineate tree crowns in a forest, and similarity in those research is that the trees have a point of maximum that allows those algorithms to fit for purpose. Peculiarly, the leaves of a banana plant are spread from its trunk, and can have multiple point of maxima. Thus, the afore-mentioned traditional methods do not fulfill our purpose.

In 2014, object-based image analysis was used to delineate potential banana plantation area in high resolution satellite images [13], to contribute to the detection of BBTV virus in Queensland, Australia. However, the objective of the research was limited not to detect individual plants, but to detect banana cultivated areas on satellite images. In contrast to the past studies, for our purpose, we use Unmanned Aerial Vehicles (UAV) for robustness in collection of aerial images from multiple altitudes. UAV provides higher temporal and spatial resolution compared to the satellite images, and also increases cost-effectiveness depending upon the study area. The scale-to-cost ratio is extremely important when it comes to small-to-medium scale farmers. This fact has led some works like [14] to use UAV for tree and nursery inventory management. [15] and [16] used UAV collected images to detect individual plants from image processing techniques based on color characteristics of trees. UAV point cloud data have also been used to detect individual trees, where they commonly calculate Canopy Height Model (CHM) and local maxima using software like *TreesVis* [17], as in [18], [19] and [20].

The shape of a banana plant on aerial images is mostly generalized by shape of a star. But in reality, it can be seen on aerial images that the shapes are irregular and uncommon from each other. Also, the overlap of adjacent plants provides severe challenges in plant counting. These challenges have been faced in the detection of palm trees over high resolution satellite images in [21] and over UAV point cloud data in [18], where they use local peak detection method to find the maximal point of the palm tree. The use of point cloud data obtained from UAV in [18] could detect 86% trees on data collected from 100m altitude, but the method performed poorly as 68% for data collected from 70m altitude. In 2014, [22] extracted ‘keypoints’ using SIFT algorithm [23], and analyzed them using a trained extreme learning machine (ELM) [24] classifier. ELM marked each palm trees by multiple keypoints, which were later merged to obtain shape of the palm trees on their data collected from UAV over a farm with well spaced cultivation with no presence of grass on the farm. A typical banana farm needs more water than palm trees and the farm is surrounded by algae and grasses. Also due to the small ‘sword sucker’ plants growing besides the originally planted banana plants (see [2] for the detailed structure of banana plants), on an aerial image it looks like multiple banana plants are overlapped together. Due to the structure of banana plant with multiple leaves growing outwards from it’s pseudostem and the existence of sword suckers, same banana plant can have multiple maxima points, which could add to the challenge of banana plant detection. Therefore, we seek an alternative to the use of point clouds or elevation models for our purpose.

In recent years, Deep Learning (DL) [25, 26] architectures have excelled the computational power of machine learning to higher degree of precision and performance by increasing the number of “layers” or “depths”. DL allows fast and automatic feature extraction from adequately large dataset, iteratively using complex models to reduce classification errors in regression [27]. DL have become core method in many researches in plant recognition [28], plant disease recognition [29, 30], weed detection [31] and crop type classification [32, 33]. A DL based oil palm tree detection was carried out on satellite images by using convolutional neural networks (CNN) [34] architecture in [35]. A review on DL in agriculture can be found in [36]. However, most of these studies are carried out on images taken in a controlled environment with proper lighting conditions. Others use satellite images as their data source, and very few used DL in UAV-collected images [37].

The irregularity in shape, lack of single peak point on young banana plants and absence of study on detection of individual banana plants on aerial images to the best of our knowledge, have led us to seek a strategic approach to automate the count of banana plants with the help of UAV, image processing methods and DL technologies. Also, DL allows re-usability of models. We apply CNN on the data variants provided by different image processing methods on aerial images of banana farm. Different image processing methods are available in regards to remote sensing applications [38], and methods like contrast stretch [39], synthetic color transformation of hue-saturation-value [40] and calculating vegetation index [41, 42] are quite significant for vegetative measures. We apply these methods to obtain multiple variants of input images, which are used to separately train a DL model based on Faster-RCNN [43] architecture, producing a different model for each variant of image processing method. Recently, Faster-RCNN is a widely used CNN architecture in agriculture ([44], [45], [46]). The results obtained from the use of CNN model over multiple variants of image processing methods are finally assembled and merged to obtain banana plant detection and count. The same process is later repeated and tested over multiple dataset collected from multiple flight altitudes, to find out better flight altitudes for young banana plants.

The rest of the paper is structured as follows: Section 2 presents the materials and method framework used to obtain our proposed algorithm; Section 3 demonstrates and discusses the experimental results; and Section 4 provides the major conclusions with potential future works.

## Materials and methods

### Dataset

A banana farm in Phra Nakhon Si Ayutthaya province of Thailand (14°19′04.5”N 100°45′18.0”E) lost some banana plants in the third month of plantation, despite proper plantation. Sixteen acres of this farm was selected as our study area, for which a flight was planned for our DJI Phantom 3 UAV. The farm was owned by King Fruits International Co., Ltd. and the data was collected under the monitoring of a staff under the company, with granted permission. High-resolution RGB aerial images of 4000x3000 pixels were collected on December 21, 2018, from 40, 50 and 60 meters above ground, according to flight plans shown in Table 1.

**Table 1:** Flight details for multiple flight altitudes.

SN	Parameters	40m	50m	60m
1	**Speed (m/s)**	10	10	10
2	**Shooting Angle**	64°	64°	64°
3	**Front lap**	75%	75%	75%
4	**Side lap**	75%	75%	75%
5	**Resolution (cm/px)**	1.78	2.03	2.54
6	**Number of Flights**	1	1	1
7	**Time of Flight**	12:00pm	11:00am	10:00am
8	**Flight Duration (minute: second)**	16:44	10:15	7:29
9	**Number of Images Collected**	398	266	182
10	**Area (acres)**	16	16	16

### Workflow for banana plant detection

**Fig 1:**
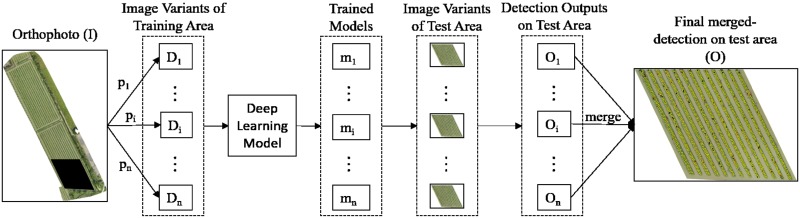
Proposed algorithm.

**Fig 2:**
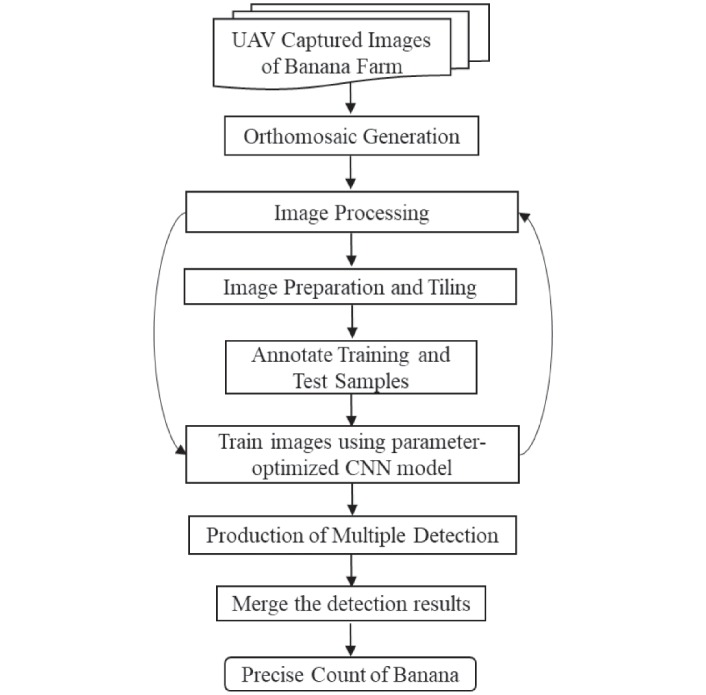
Workflow of proposed method.

In this paper, we propose a strategic algorithm to count the number of banana plants in aerial images. Let us assume a training dataset (*D*) = an orthomosaic (*I*), with *k* number of training samples of banana plants. Then {*D*_1_, …, *D*_*i*_, … *D*_*n*_} are variants of the orthomosaic, which are obtained by using different processing methods {*p*_1_, …, *p*_*i*_, …*p*_*n*_} on the original training dataset *D*. Each variant *D*_*i*_ is then tiled into smaller image tiles to extract training samples needed to train a parameter-optimized DL model, thus producing trained detection models {*m*_1_, …, *m*_*i*_, …*m*_*n*_}. Model *m*_*i*_ is then used to detect banana plants image variant *D*_*i*_ of a separate validation area (any other part of farm except the training area), to produce multiple detection outputs {*O*_1_, …, *O*_*i*_, …, *O*_*n*_}. These *O*_*n*_ detection outputs are then merged to get the final detection output *O* of *I*. A schematic diagram of the algorithm is shown in Fig 1, and to perform this algorithm to our problem of counting banana plants, we use the workflow as shown in a schematic diagram of Fig 2. It is to be noted here that merging results obtained on multiple flight altitudes is not a part of the algorithm. We separately repeat the same algorithm, with some change in parameters, to test the overall method on different dataset provided obtained from multiple flying altitudes.

The first step after collecting images is to create an orthomosaic (*I*) with RGB bands. This RGB orthomosaic is not sufficient to provide enough variance in data that is required to train a DL model for precise detection of banana plants. Also the irregularity in shape of banana plants, effect of shadow, angle of reflection of light to the camera adds to this challenge. This is why we need to exploit the low frequency information in the orthomosaic, which is the vegetative properties of banana plants in our dataset. Therefore, we process the orthomosaic using various image processing methods (*p*_*n*_), to exploit the vegetative properties of banana plants, and produce different variants of orthomosaic (*D*_*n*_). From each *D*_*i*_, we separate the training area and region of interest (ROI) (i.e validation area), to test our final CNN model obtained after training. For both training and validating area, the large-sized images are then tiled to smaller size of 600x600 pixels.

After image preparation by tiling, next step is to carefully annotate banana plant samples seen on the image tiles of training area, by manually drawing bounding boxes of rectangular shape around the plants. Annotation of banana plants completely depends on human interpretation. The annotated image tiles of each *D*_*i*_ are then used separately, to train and test CNN based parameter-optimized model for each *D*_*i*_, producing *n* number of ready-to-use trained CNN model for each *p*_*n*_ variant. These trained models can now be used to test the image tiles of each *p*_*n*_ of our validation area, to produce *n* sets of detection results of ROI. These multiple result sets are then finally merged to obtain final detection results. The step-wise workflow of our algorithm is presented below, where we show some sample results for the image tiles produced from orthomosaic generated from the images collected from 40m altitude.

All processing steps of this research are performed in a computer with following specifications:

- *CPU*: Intel(R) Core(TM) i7-7700HQ CPU @ 2.80GHz.
- *RAM*: 24GB.
- *GPU*: NVIDIA GeForce GTX 1050 Ti (Driver: 23.21.13.9125).
- *OS*: Windows 10 Education, 64-bit.

### Orthomosaic generation

After collecting images, first step of post-processing UAV aerial images in photogrammetric mapping is to stitch together the overlapping image pairs. We used a state-of-the-art software called Pix4Dmapper [47], which is capable of adjusting geometric and radiometric effects, extract pixels on the images that share common view to produce 3D point clouds [48], which can be used to create texture model, and finally project them into an orthomosaic (aka. orthophoto). We generate orthomosaic for each image dataset collected from multiple altitudes. For simplicity, lets call these multiple variants of orthophoto due to flight altitude as *altitude variants*. High-resolution orthomosaic obtained from Pix4d for altitude variants of our study area were produced with the details shown in Table 2.

**Table 2:** Orthomosaic processing details for altitude variants.

SN	Parameters	40m	50m	60m
1	**Processing Time (minutes)**	209	143	92
2	**Number of batch**	1	1	1
3	**Orthomosaic Width**	23655	19142	16297
4	**Orthomosaic Height**	34390	26878	23055

### Image processing

After generating orthomosaic *I*, the next step is to use image processing methods to obtain multiple variants *D*_*n*_ of *I*. The reason to use these methods was figured out once we produced initial results based running CNN model on normal RGB images, in which the count of banana plant was considerably low. It was learnt that, edge detection being the fundamental step of CNN, DL needs objects (banana plant) to be properly distinguished from background, to make the objects distinct and produce better results. In our case, banana farm has grasses covering the soil and the color of banana plant and grass is almost similar. Also, the shadow of top leaves blocks lower leaves of the plants, making their shape seem irregular from zenith. Another reason for lower number of plants being detected is that CNN models require large number of training data and more of variations in images. The azimuth of the sun, reflectance angle of light and height of UAV camera adds to the challenge. A solution to these problems is to separately apply different image processing methods (*p*_*n*_), and separately train CNN models on processed image variants (*D*_*n*_) to produce optimized inference models from each set of uniquely processed images. The results (*O*_*n*_) obtained from different inference models can be finally merged to obtain better detection.

There exists number of methods for image enhancement. Since our target is to detect banana plants, we need the methods to enhance the low frequency information of vegetation that are normally dominated by high frequency information in images. We explored methods that can enhance vegetative properties on RGB aerial images in canopy scale, and found three methods: Linear Contrast Stretch (*p*_1_), Synthetic Color Transform (*p*_2_) and Triangular Greenness Index (*p*_3_), to enhance color histogram, radiance values and vegetative properties in orthomosaic image, which are explained below.

#### Linear contrast stretch

Each pixel of an aerial image consists of radiance value from real scene, which is converted into range of 0 to 255 to store in the image. These converted numbers are called Digital Numbers (DN). However, the range of radiance value is generally less than the full range of DN. Due to this difference in available range and actual range, images have low contrast and do not use the full range of display. In contrast enhancement [49], these DN numbers are transformed into Gray Levels (GL) display space, using a mapping function, to improve visual quality of image. This transformation of DN into GL such that DN range fills available GL range, is called *Contrast Stretch*. Similarly, one way is to fill the display range of [0, 255] from minimum DN to maximum DN of image, and this is one of the methods of *Linear Contrast Stretch* (abbr. LCS).

**Fig 3:**
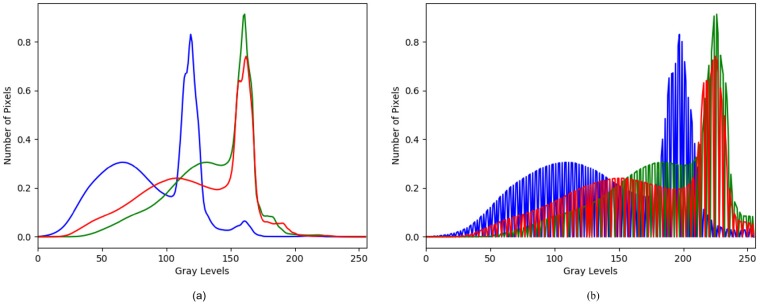
RGB color histogram. (a) Color histogram of orthomosaic (b) Color histogram of orthomosaic after LCS.

We use *saturation stretch* of 1% with LCS to obtain greater increase in contrast. In this approach, min-max range of DN is made to fit to the range of GL i.e. [0, 255], and 1% of upper and lower extremes of DN range are “saturated” (clipped). This exploits the radiance values by brightening the pixels of banana plants and their leaves in our image. Fig 3 shows RGB color histogram of original and contrast stretched orthomosaic for 40m altitude variant.

#### Synthetic color transform

Synthetic Color Transform (abbr. SCT) is a process to assign synthetic colors to a gray scale image or a single band of image. One of the ways to do it is to transform hue, saturation and value (HSV) data of an image into RGB color space, such that hue and value are assigned with the low frequency and high frequency information [40]. The saturation can be fixed to some level. We used the green band of our orthomosaic to transform into synthetic colors. Initially, a single band of an image is a gray scale image. High pass and low pass filter was used to extract the high and low frequency information respectively. And the saturation was fixed to 0.5. SCT is useful to see the low frequency information present due to scattering of surface from vegetation, that are dominated by high frequency information in an image.

#### Triangular greenness index

Another method of image processing that we use is to calculate Vegetation Index (VI). To understand which VI to use, we need to keep in mind that we are using a digital camera with RGB bands, and near-infrared (NIR) band is not available in our images. The plant we are focusing on is made of large leaves of green color, surrounded by green grasses, which makes it difficult for computer vision techniques and human eyes to understand the difference between grass and banana leaves due to minimal difference in chlorophyll content of grass and banana leaves. We need a VI that is sensitive towards the change in the difference in leaf chlorophyll content of leaves in canopy scale, as the UAV flies in lower altitude during data collection. Triangular Greenness Index (TGI) was developed to solve this problem in [50] for digital cameras with RGB bands, and hence is the best-fit VI for our purpose.

TGI derives chlorophyll content in leaves in canopies by calculating the area of triangle formed by three points: (480nm, *R*_480_), (550nm, *R*_550_), and (670nm, *R*_670_) as shown in Eq 1. In [50], authors used spectral reflectance and wavelength of Red (670), Green (550) and Blue (480) bands of Landsat Thematic Mapper. They also suggested the use of RGB bands for digital camera instead of narrow bands. For a typical CMOS camera, the wavelengths are normalized by green bands in [51], making TGI as in Eq 2, which was used to calculate TGI on our orthophoto with *R*_*Red*_, *R*_*Green*_ and *R*_*Blue*_ being the radiance values on RGB bands.

$$\begin{array}{c} \begin{array}{c} TGI=-0.5*[190*\left(R_{670}-R_{550}\right)-120*\left(R_{670}-R_{480}\right)] \end{array} \end{array}$$ (1)

$$\begin{array}{c} \begin{array}{c} TGI=R_{Green}-0.39*R_{Red}-0.61*R_{Blue} \end{array} \end{array}$$ (2)

The green leaves of banana plant have significantly high chlorophyll content, and similarly the greenish-yellow and yellow-greenish leaves (mature banana plant leaves) have lower chlorophyll [52]. The young banana plants in our field has green leaves. TGI brightens and highlights the pixels with green banana leaves, having high chlorophyll content in our aerial image. After calculation of TGI, the RGB image input gets converted into an 8-bit image.

### Image preparation and tiling

**Fig 4:**
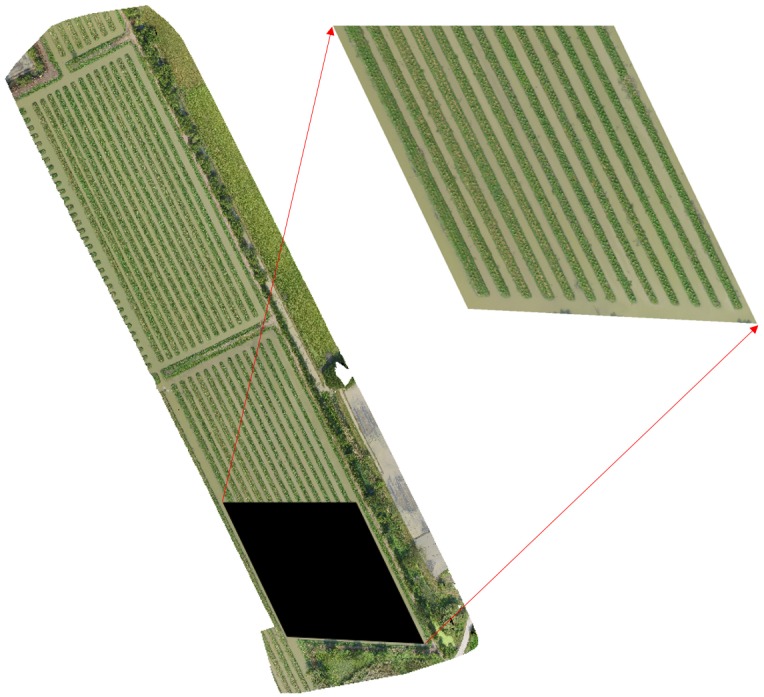
Study area separated into training region and ROI (top right).

After applying the three image processing methods *p*_1_, *p*_2_ and *p*_3_, we obtain three image variants, *D*_1_, *D*_2_ and *D*_3_, to train and test our DL model. The training and ROI area is now separated from each *D*_*i*_ as shown in Fig 4. Our ROI has 2695 banana plants in ground truth (GT), that needs to be counted.

After separating ROI, image formats are converted to decrease the computational expense of work. The three image processing variants of training area are of *tiff* file format and are of same dimension as original orthomosaic. The tiff images are difficult to decode and larger images occupy heavy amount of memory and Graphic Processing Unit (GPU) of a computing machine, making DL computationally expensive. Therefore, we convert *D*_1_, *D*_2_ and *D*_3_ into smaller tiles of Joint Photographic Experts Group (JPEG) format, to minimize the file size without loss in spectral data.

The training area orthomosaic was tiled into 600x600 pixel tiles, in a grid fashion without using strides, padding or overlap. For 40m data, we produced 2116 image tiles of 600x600 pixels from training area, out of which, only 458 tiles consisted of atleast a single banana plant. These 458 tiles were manually separated into two sets of images: *train tiles* (366) and *test tiles* (92). Similarly, the details for other altitude variants are shown in Table 3.

**Table 3:** Training details of altitude variants.

SN	Parameters	40m	50m	60m
1	**Total training area tiles**	2116	1300	1004
2	**Training Area Usable Tiles**	458	365	230
3	**Train Tiles**	366	292	184
4	**Test Tiles**	92	73	46
5	**ROI Tiles**	247	165	108
6	**Overlap for ROI tiles (pixels)**	50	50	50
7	**Total Samples in Training Area**	7212	7310	6101
8	**Training Samples**	5770	5818	4803
10	**Test Samples**	1442	1492	1298
11	**Train Time (RGB) in minutes**	90	303	206
12	**Train Time (LCS/D1) in minutes**	129	252	229
13	**Train Time (SCT/D2) in minutes**	180	281	180
14	**Train Time (TGI/D3) in minutes**	188	344	219
15	**Loss (RGB)**	0.012	0.005	0.015
16	**Loss (LCS/D1)**	0.007	0.013	0.014
17	**Loss (SCT/D2)**	0.025	0.020	0.005
18	**Loss (TGI/D3)**	0.037	0.002	0.011

We used “overlap” of 50 pixels to tile ROI into “ROI tiles”, such that the plants at the edges of tiles would not be missed later in the results. This overlap can be of any number as long as we merge the multiple detection of same plants later in our approach, but it is equally important to save the computational time. A single young banana plant in 40m orthophoto covered less than 50x50 pixel area, which made us choose the value of 50 for the overlap. The difference in detection results with and without using this overlap is discussed later in results section below. 247 ROI tiles of 600x600 pixels were produced to validate the final CNN model. The naming of the tiles was done as {tile_0_0, tile_0_550, …, tile_550_0, tile_550_550, …, tile_*x*_*i*__*y*_*j*_}, so that the tile number information of ROI tiles can later be used to merge them.

**Fig 5:**
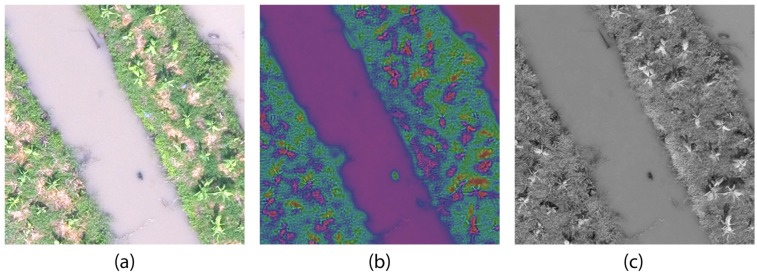
Sample image tiles of 40m altitude variant. (a) LCS (*p*_1_), (b) SCT (*p*_2_) and (c) TGI (*p*_3_).

The train tiles and test tiles of each image variant *D*_*i*_ can now be used to train parameter-optimized DL to produce frozen model *m*_*i*_, and the tiles from ROI can be used to obtain detection result *O*_*i*_ for evaluation. A sample image tile of *D*_1_, *D*_2_ and *D*_3_ of 40m are shown in Fig 5(a), 5(b) and 5(c) respectively.

### Train images using parameter-optimized CNN model

This step deals with the detection of banana plants using DL model, and is a crucial step in our approach. The set of image tiles produced for each variant of training area are now separately used to train DL model to produce different sets of outputs *O*_1_, *O*_2_ and *O*_3_ for our ROI. For detailed clarification, we break the tasks performed to use DL on our image dataset, into two sub-steps below.

#### Image annotation

The image tiles obtained in previous step are manually annotated by drawing rectangles to sufficiently surround the objects. Total 7212 samples of banana plants were manually annotated for the train and test tiles for 40m image tiles, out of which 5770 training samples and 1442 test samples were produced from 366 and 92 train and test tiles, respectively. The manually annotated training and test samples were then forwarded to train a CNN model mentioned below. The details on other altitudes is given in Table 3.

It can be seen that the number of samples for different altitudes are different. One of the main reason for this is the difference in object size in altitude variants. The focal length of camera we used in our UAV was 4mm. Therefore, the photographic scale of images collected from 40, 50 and 60m altitude were 1:10000, 1:12500 and 1:15000 respectively. To simplify, if a banana plant occupies 9*m*^2^ in ground truth, it occupies 0.09, 0.06 and 0.04 *mm*^2^ on the altitude variants of 40, 50 and 60m respectively. So the object size gets smaller as flight altitude increases. Also due to factors like resolution of images, side lap and front lap during flight plan, calibrations and stitching during orthomosaic generation, wind during flight, shadow of the plants, azimuth of sun, etc., same plant can be distorted or blurred in different altitude variants. These distorted part of image and the plants that are too small, are not good enough to train the models because of the risk of increasing incorrect detection, and we do not annotate these misleading images of banana plants. For our 40m dataset, it was observed during annotation that, the orthophoto was distorted for the left most cultivar of our farm. The banana plants on this part of orthophoto were therefore not annotated, making the number of training samples for 40m variant smaller than 50m variant. But for 60m, the banana plants that were confusing and very small were ignored during annotation, making training sample for 60m the least.

#### CNN training and parameter optimization

Now we use the annotated images consisting of training and test samples of our training area to train an object detection method that is based on CNN architecture. Out of many available models, we chose Faster-RCNN for our purpose as it is in state-of-the-art performance. The architecture of Faster-RCNN is shown in [43], which works in two components: a Region Proposal Network (RPN), that proposes region of object location on the images, and a CNN network to classify objects in those proposed regions. An input image is first processed by Faster-RCNN with a feature extractor to produce feature maps. A feature map is a network of CNN consisting of a convolution layer and a pooling layer. The feature map is then passed to RPN for region proposal. Unlike the original Faster-RCNN that used CNN models like ZFNet [53] and VGG-16 [54] as feature extractor, we used 42-layered Inception-v2 model (see [55] for detailed architecture), which achieved state-of-the-art in ILSVRC 2014. The RPN then scans the feature maps by sliding windows to check whether an object (banana) exists or not. As the sliding windows are of fixed-size, multiple anchor boxes of varying scales and aspect ratios are used, to deal with dissimilarity in the shape of objects. We used 12 anchor boxes of 4 different scales and 3 different aspect ratios for our purpose. By default, RPN proposes multiple regions for same object, and they become superfluous. Therefore, the number of regions were limited to 300 using non-maximum suppression. The variation in sizes of these regions were set to fixed-size using a Pooling Layer, and then passed to a fully connected layer with SOFTMAX score converter for the calculation of bounding box regression loss and object classification loss, as originally suggested by [56] and [43].

We used Tensorflow Object Detection API [57]—a DL framework in Python language—to implement the Faster-RCNN. We took the pre-trained model of Inception-v2 trained in Common Objects in Context (COCO) image dataset, and optimized the configuration parameters mentioned in previous paragraph, despite the unavailability of literature on parameter-optimization for banana plants. Also, data augmentation methods like random horizontal and vertical flip were activated to randomly augment the image tiles during the training, to provide variability in object samples. Data augmentation provides variability during the learning for DL models, and parameter optimization increases the performance of DL, thus increasing the number of true positives (TP), while decreasing false positives (FP) and false negatives (FN) of the objects detected.

During the training, loss function for bounding box regression and object classification was carefully monitored, and the training was continued until the loss decreased to less than 0.05 for variant *D*_*i*_. Stopping the training at minimum loss, the models were frozen at the corresponding iteration step, and saved as frozen detection model *m*_*i*_ for each *D*_*i*_. These models can be now used to detect banana plants on ROI tiles of corresponding image processing variants, to obtain detection outputs *O*_*i*_. Table 3 shows the number of images produced in each steps of image preparation and tiling, number of training and test samples, train time and loss of models *m*_1_, *m*_2_ and *m*_3_ for altitude variants.

### Production of multiple detection results

The three models are now used to test and produce detection results (*O*_1_, *O*_2_ and *O*_3_) over the three sets of ROI tiles. While running each *m*_*i*_ into ROI tiles, intersection over union (IoU) threshold was set to be 0.5, instead of default and generally used value of 0.8. IoU measures the accuracy of detection by calculating the ratio of area of overlap between two bounding boxes to the area of union. IoU threshold ranges from 0 to 1, and decreasing it results higher recall and lower precision in detection, and vice-versa. It makes sense to lower this threshold at this step, because in the next step, we merge the detected multiple bounding boxes into a single box anyways.

**Fig 6:**
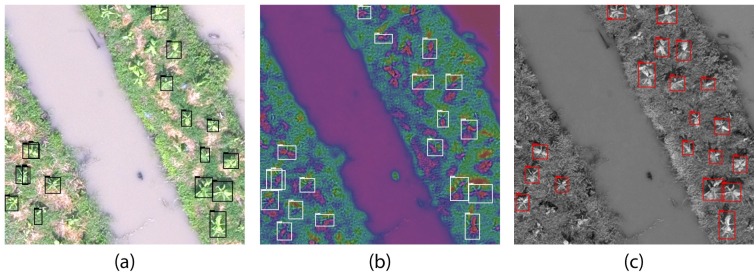
Detection results on variants of image processing methods on a ROI tile of 40m. (a) LCS (*p*_1_), (b) SCT (*p*_2_) and (c) TGI (*p*_3_).

Fig 6(a), 6(b) and 6(c) shows a sample tile of *O*_1_, *O*_2_ and *O*_3_ for 40m, where it can be seen that 15, 18 and 19 out of 24 plants are respectively detected. Some small plants and plants that are closely occluded to other plants are not detected. Also there exists a problem of multiple detection of same object in some tiles, as seen in Fig 6(a) and 6(b), which is due to the lowering of IoU threshold. This problem is solved while merging the results in next step.

### Merge the detection results

Now we combine the results of *O*_1_, *O*_2_ and *O*_3_ to increase the count of banana plants and to solve the problem of multiple detection of same plant as seen in the sample before, in the following four steps:

1. Collect bounding boxes of detection from each ROI tiles.
2. Calculate centroid of each bounding box.
3. Add the tile number information on x and y-value of centroids to overlay them on original ROI image.
4. Merge the centroids that are clustered within some threshold of *Euclidean Distance*.

**Fig 7:**
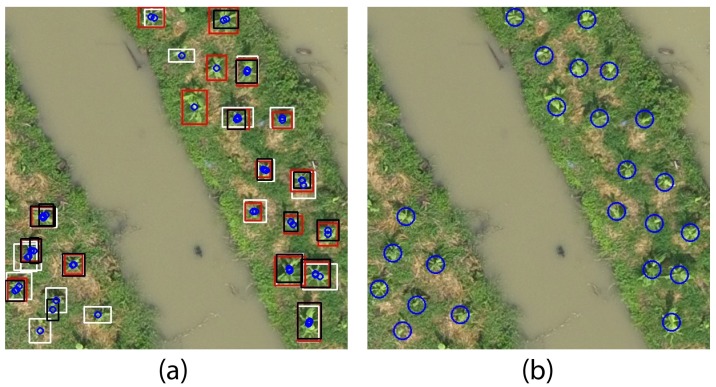
Merging detection results on a sample ROI tile of 40m. (a) Bounding boxes from LCS (Black), SCT (White) and TGI (Red), and their centroids (small blue circles) on a sample image tile of ROI. (b) Merged centroids by taking euclidean distance threshold of 30 pixels are represented by blue circles of 15px radius.

The threshold is estimated by taking a value less than the euclidean distance between the closest plants in image. For 40m, 50m and 60m altitude variants, we used 30, 25 and 20 pixels of threshold respectively. Fig 7(a) shows the result of first and second step for a sample ROI tile of 40m, and Fig 7(b) shows how the merged output would look like on the sample tile, without following third step of merging (for demonstration).

It can be seen that, after merging the results, total detection has increased to 23 out of 24 plants, in our sample tile. This is because of decreased false negatives (FN) due to combination of multiple detection results. It can be seen in Fig 7(a) that some plants are detected in only one variant of image processing method. The merged detection results on sample ROI tile in Fig 7(b) shows that the detection of plants can be increased by enhancing the vegetative property of aerial image, using methods like contrast stretch, vegetation index and HSV color transform.

**Fig 8:**
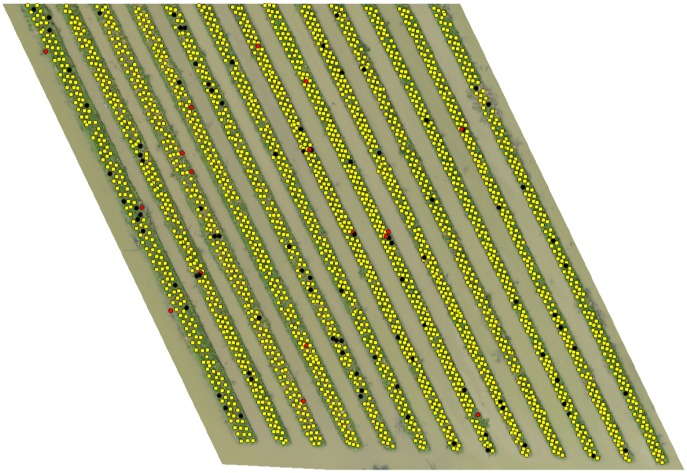
Final detection on ROI of 40m altitude variant.

**Fig 9:**
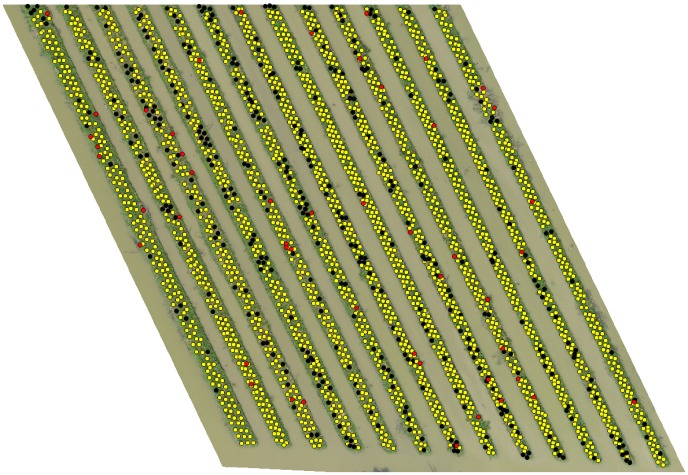
Final detection on ROI of 50m altitude variant.

**Fig 10:**
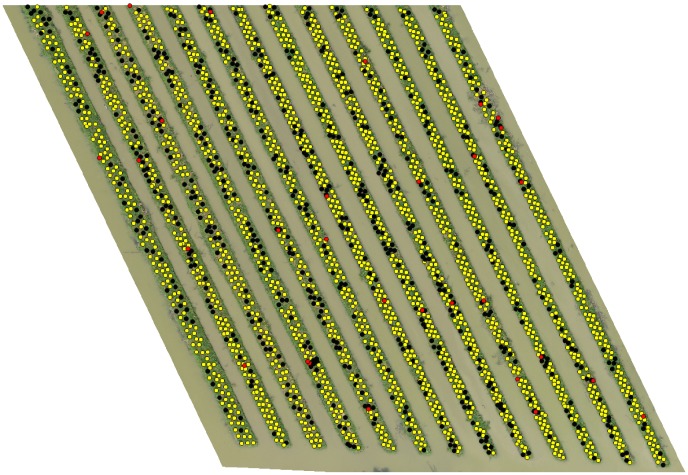
Final detection on ROI of 60m altitude variant.

Figs 8, 9 and 10 shows the final detection result obtained after the overall algorithm on orthomosaic (*I*) of 40m, 50m and 60m altitude variants respectively. The yellow, red and black markers represent the correct, incorrect and missed detection respectively. In next section, we evaluate the performance of our algorithm to detect banana plants on all altitude variants and see what what happens if we combine the detection results of all altitude variants.

## Results and discussion

In this section, we evaluate quantitative and qualitative performance of our algorithm on our ROI with 2695 banana plants. We investigate the performance of detection results on each variants provided by image processing methods, and also discuss results on the altitude variants. To calculate the overall accuracy of our algorithm in ROI, we calculate precision and recall of banana plant detection to compare against ground truth. Eqs 3, 4 and 5 shows the formula for precision, recall and overall accuracy, where a correct detection is a case where a bounding box obtained from detection result sufficiently or insufficiently contains a single banana plant. We use these formulas to discuss the difference in performance due to different image processing methods, and also discuss the performance of our algorithm over multiple altitude variant dataset.

$$\begin{array}{c} \begin{array}{c} \text{Precision}=\frac{\text{Total}\;\text{number}\;\text{of}\;\text{correct}\;\text{detection}\;\text{of}\;\text{plants}}{\text{Total}\;\text{number}\;\text{of}\;\text{all}\;\text{detected}\;\text{objects}} \end{array} \end{array}$$ (3)

$$\begin{array}{c} \begin{array}{c} \text{Recall}=\frac{\text{Total}\;\text{number}\;\text{of}\;\text{correct}\;\text{detection}\;\text{of}\;\text{plants}}{\text{Total}\;\text{number}\;\text{of}\;\text{plants}\;\text{in}\;\text{ground}\;\text{truth}} \end{array} \end{array}$$ (4)

$$\begin{array}{c} \begin{array}{c} \text{Overall}\;\text{Accuracy}=\frac{\text{Precision}+\text{Recall}}{2} \end{array} \end{array}$$ (5)

### Performance of image processing methods on altitude variants

The idea of using multiple image processing methods to exploit vegetative properties of RGB image, train separate DL models and later merge the detection results, was composed after we first got the detection results on normal RGB orthophoto. Tables 4, 5 and 6 shows the recall, precision and overall accuracy of our models on RGB images and the other three image variants—LCS, SCT and TGI—for three of our altitude variant datasets of 40m, 50m and 60m respectively. It can be seen in these results that the DL model trained on RGB images produces poor results, which is why we produce detection results on LCS, SCT and TGI image variants, and merge the results. This increased our accuracy measures to upto 97.9% overall accuracy for 40m dataset as seen in Table 4.

**Table 4:** Detection performance on ROI of 40m altitude variant.

Image Processing Method	Correct Detection	All detected Objects	Recall	Precision	Overall Accuracy
**RGB**	2123	2124	0.788	1.000	0.894
**LCS**	2217	2229	0.823	0.995	0.909
**SCT**	2335	2354	0.866	0.992	0.929
**TGI**	2372	2373	0.880	1.000	0.940
**Merged**	2598	2615	0.964	0.993	0.979

**Table 5:** Detection performance on ROI of 50m altitude variant.

Image Processing Method	Correct Detection	All detected Objects	Recall	Precision	Overall Accuracy
**RGB**	1893	1904	0.702	0.994	0.848
**LCS**	1930	1933	0.716	0.998	0.857
**SCT**	1955	1990	0.725	0.982	0.854
**TGI**	1896	1899	0.704	0.998	0.851
**Merged**	2294	2343	0.851	0.979	0.915

**Table 6:** Detection performance on ROI of 60m altitude variant.

Image Processing Method	Correct Detection	All detected Objects	Recall	Precision	Overall Accuracy
**RGB**	1232	1233	0.457	0.999	0.728
**LCS**	1308	1313	0.485	0.996	0.741
**SCT**	1626	1656	0.603	0.982	0.793
**TGI**	1483	1491	0.550	0.995	0.772
**Merged**	2043	2074	0.758	0.985	0.872

At first, we had trained the Faster RCNN Inception V2 model on our training area to create three trained-models for each image variants, without using overlap during the tiling process of ROI. The results was 0.728, 0.660 and 0.419 recall for 40m, 50m and 60m variant respectively. Later we modified our tiling step for ROI by using overlap of 50 pixels as explained previously, which allowed the detection of banana plants at the edges of ROI tiles. This increased the recall to 0.788, 0.702 and 0.457 for 40, 50 and 60m altitude variant respectively. The detection results obtained from all methods on ROI of 40m, 50m and 60m are shown in Tables 4, 5 and 6 respectively.

Aside from the results on normal RGB images, the detection results shown by all other image processing methods LCS, SCT and TGI are much better. The LCS method stretched the contrast of images, allowing to detect smaller plants that were missed in normal RGB images. This is due to the stretch of GL value range in the images. The bright pixels became vibrantly brighter and the dark pixels even darker, separating pixels covering grass from the pixels covering bright banana leaves. Furthermore, SCT could detect the plants that were missed by LCS, with higher performance in detection. This is because SCT emphasizes the low-frequency information that are present due to surface scattering difference from vegetation on the ground. Additionally, TGI brightened the high chlorophyll containing green leaves of young banana plants, which allowed to detect the plants with high precision.

If we take a look at the precision of each method’s detection, we can see that all of them has achieved over 98% despite using only 0.5 IOU threshold during CNN model configuration. The precision of the DL models alone would not produce this level of precision if we did not merge the detection bounding boxes using Euclidean Distance method. All in all, the merged results on LCS, SCT and TGI combined their individual detection results which increased the recall to 96.4%, 85.1% and 75.8%. The overall accuracy is even more higher because of better precision in detection. Comparing these results to the initial results on normal RGB images, we can conclude that our overall algorithm to detect banana plants using multiple image processing techniques like LCS, SCT and TGI that exploits the vegetative properties of images, is efficient in terms of performance.

The performance of object detection in CNN is directly affected by object size, and this can be seen in the results. Also, close observation of results showed that, with the increase in flight altitude, two plants that were close together were detected as a single plant in higher altitude variants of ROI. This decreased the precision of detection for 50m and 60m variants, also lowering the overall accuracy. From Tables 4, 5 and 6, we can say that, lower the flight altitude gets, or higher the resolution of image, better is the detection of banana plants. For us, 40m is the best altitude for banana plant detection among the three because of several reasons. One, the whole image dataset of 40m was taken in a single flight, orthophoto was generated in a single batch of processing, and the detection results using this dataset was the best among our altitude variants. Decreasing the altitude further might increase the number of UAV flights because commonly used UAV’s like DJI Phantom are equipped with batteries that lasts around 20 minutes for flights.

In the next section, apart from our original algorithm, we combine the detection results of our altitude variants to see how the combination of altitude variants will affect the performance of detection.

### Combining the results of multiple flight altitudes

The 60m, 50m and 40m images were collected at 10am, 11am and 12pm on a bright sunny day. The shape of young banana plants being irregular and smaller, some plants would be missed on one flight altitude than other, because of the factors like azimuth of the sun, shadow of the plant, reflection angle of light on plants, wind during flight, presence of grasses on the farm, side overlap and front overlap during flight plan and corrections during orthomosaic generation. Therefore, some plants missed by 40m altitude are detected by 50m or 60m altitude variants. Eventhough the detection results of 40m have already shown 96.4% of recall and 97.9% overall accuracy, it still makes sense to increase the detection performance by combining results of altitude variants. Since the altitude variants were collected at different time of the day, it would make more sense to say that these altitude variants increases the temporal resolution of our study.

To merge the detection results of altitude variants, we first linearly transform the scale of centroids of bounding boxes of higher altitude variants (50m and 60m) to the dimension size of lower variant of 40m. The new *x* and *y* value of the centroids are calculated using using Eqs 6 and 7 respectively, where the values of transform parameters are taken from Table 7. The width and height of ROI images for {40m, 50m, 60m} are {10032, 7877, 6469} and {7048, 5534, 4545} respectively, which decides the value of *x*_*scale*_*factor*_ and *y*_*scale*_*factor*_. The *x*_*shift*_ and *y*_*shift*_ are the lateral shifts of objects in different images that needs to be adjusted, which were observed from manually trying to overlay the transformed centroids over the center of corresponding banana plants in 40m image.

$$\begin{array}{c} \begin{array}{c} x_{scaled}=\left(x*x_{scale\_factor}\right)+x_{shift} \end{array} \end{array}$$ (6)

$$\begin{array}{c} \begin{array}{c} y_{scaled}=\left(y*y_{scale\_factor}\right)+y_{shift} \end{array} \end{array}$$ (7)

**Table 7:** The transform parameters.

SN	Parameters	50m to 40m	60m to 40m
1	*x*_*scale*_*factor*_	10032/7877	10032/6469
2	*y*_*scale*_*factor*_	7048/5534	7048/4545
3	*x*_*shift*_	-81.996	57.996
4	*y*_*shift*_	38.003	-37.004

The transformed centroids of detection of bounding boxes of higher altitude variants were collected and overlaid on ROI of 40m. Then the closely clustered centroids were merged using euclidean distance threshold of 30 pixels (as before in merging of detection of multiple image processing methods), to avoid redundant detection.

**Fig 11:**
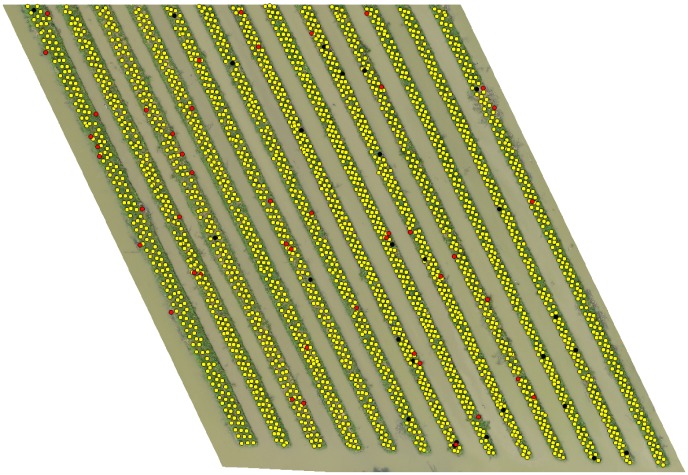
Detection results of combination (40+50)m.

**Fig 12:**
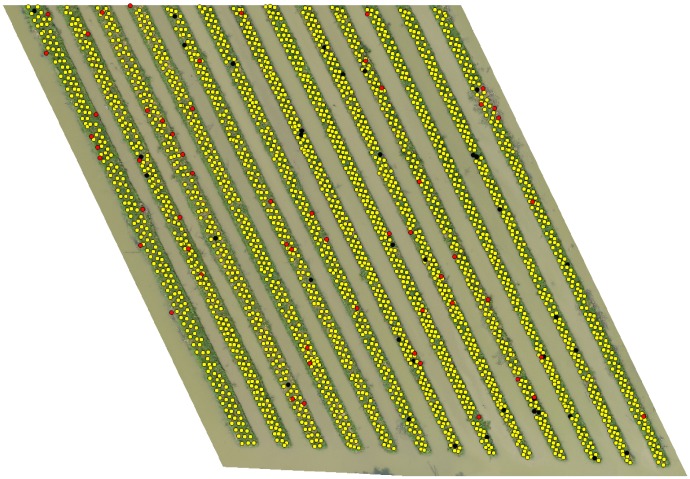
Detection results of combination (40+50+60)m.

Table 8 shows the final detection results of individual altitude variants and combination of (40+50)m and (40+50+60)m, which shows the first combination produced better detection results (99% recall) than the later one (98.6% recall) for our image dataset. This is because the (40+50+60)m combination also merged the poor results of 60m variant. The detection results of (40+50)m and (40+50+60)m combination are shown in Figs 11 and 12 respectively, where yellow, red and black markers represents the correct, incorrect and missed detection respectively. The computation cost and time of collecting multiple altitude variants and combining them is much more higher. But the use of UAV have allowed to do increase the temporal resolution and is still much more efficient than working on satellite images. Combining the results of two altitude variants, in our case 40m and 50m, have shown significant increase in detection and count of banana plants, and the execution of combination of two altitude variants will still take less time than all variants. Therefore, we would like to suggest that use of two proper altitude variants (40m and 50m in our case), is better for detection and counting of plants on UAV-collected aerial images, but leave the idea open to the readers.

**Table 8:** Detection performance after combination of altitude variants.

Altitude Variants	Correct Detection	All detected Objects	Recall	Precision	Overall Accuracy
**40m**	2598	2615	0.964	0.993	0.979
**50m**	2294	2343	0.851	0.979	0.915
**60m**	2043	2074	0.758	0.985	0.872
**(40+50)m**	2668	2714	0.990	0.983	0.987
**(40+50+60)m**	2658	2715	0.986	0.979	0.983

## Conclusion and future works

This research developed an algorithm to precisely detect and count the number of banana plants in a farm. For demonstration, high-resolution RGB images were collected using UAV to produce orthomosaic of the study area having more than 10 thousand young banana plants. As the parameter-optimized DL models could not produce desired accuracy from RGB orthomosaic alone, three image processing methods were chosen to enhance the low frequency vegetative properties of orthomosaic and produce multiple variants of images. We trained a Faster-RCNN Inception-V2 model separately on the images obtained from each image processing method’s variant. These trained models were then used to detect banana plants on corresponding image variants of ROI, producing multiple detection results, which were finally combined to get the final detection and count. The overall algorithm could produce upto 97.9%, 91.5% and 87.2% of overall accuracy on three of our altitude variant dataset of 40m, 50m and 60m respectively.

The research also discussed on appropriate altitude to collect images, among the three variants, and also discussed the results of combining the detection results obtained from multiple altitude variants. For the 40m dataset, which was collected in a single flight of less than 17 minutes for our 16 acres study area, the algorithm produced overall accuracy upto 97.9%. We therefore suggest that 40m is a proper flight altitude for similar works, using mainstream and cheaper UAV’s like DJI Phantom series whose batteries normally lasts for 20 minutes. Also for anyone who is concerned about increasing the precision of plant detection, can take two flights and combine the produced results later. The combination of detection results from 40m and 50m dataset could correctly count upto 99% of banana plants on our ROI. However, this combination increases the time for computation and data collection, which is why we would like to leave the idea open to the readers.

This algorithm can be further used in other farms to detect and count banana plants, and the strength of detection can be increased even more by training the CNN model in larger dataset with multiple crops, which will be our future work. Also an alternative solution to combine the detection results and an algorithm to run the DL models on large images with less computational expenses should be sought out for remote sensing applications. The implementation of this research will help the farmers to estimate their production of banana, and also help to map the location of farm that needs to be re-cultivated, furthermore increasing the production.
